# Patient-reported outcomes after ipsilateral radiation therapy for N2b tonsillar squamous cell carcinoma

**DOI:** 10.1007/s12672-025-03896-z

**Published:** 2025-11-25

**Authors:** Chike O. Abana, Adam S. Garden, G. Brandon Gunn, Gregory M. Chronowski, Abdallah S. R. Mohamed, Andrew J. Frankart, Natalie Geier, Houda Bahig, Carly E. A. Barbon, Kate Hutcheson, Vinita Takiar, Clifton D. Fuller, Steven J. Frank, David I. Rosenthal, Jack Phan

**Affiliations:** 1 Department of Radiation Oncology, The University of Texas MD Anderson Cancer Center, Houston, TX 70030 USA; 2 Department of Radiation Oncology, University of Cincinnati, Cincinnati, OH 45219 USA; 3 The Christ Hospital, Cincinnati, OH 45221 USA; 4 Centre Hospitalier de L’Université de Montréal, Montreal, QC H2X 3E4 Canada; 5 Speech Pathology and Audiology, Department of Head and Neck Surgery, The University of Texas MD Anderson Cancer Center, Houston, TX 70030 USA

**Keywords:** PRO, Toxicity, Unilateral radiation therapy, MDASI, MDADI, MD Anderson symptom inventory for head and neck, HPV, Xerostomia

## Abstract

**Background:**

Previous studies have reported excellent disease control and survival in patients with well-lateralized, American Joint Committee on Cancer (AJCC)-7 T1-2N2b tonsillar squamous cell carcinoma (SCC). The reduced treatment volume is associated with lower rates of physician-assessed toxicity. Patient-reported outcomes (PROs) have been proposed as a similarly reliable measure, but the body of literature is limited for unilaterally treated patients. Our goal was to review PROs of such patients who had reduced treatment volumes.

**Methods:**

We reviewed PROs of patients with AJCC-7 T1-2N2b disease treated with ipsilateral radiation therapy (RT), with or without surgery or chemotherapy before RT. PROs were measured using the MD Anderson Symptom Inventory for head and neck cancer after a median of 28.9 months.

**Results:**

Forty-eight patients were included in the study: 36 (75%) had human papillomavirus-positive disease, 20 (42%) had ≥ 2 nodal levels involved, 15 (31%) had extranodal extension and all patients had N2b disease. Most patients reported no symptoms; a median 72.9% for all 10 head-and-neck symptoms, 75.0% for all 12 core symptoms, and 83.3% for all 6 interference symptoms reported a score of “0”. The most common head-and-neck and core symptoms were dry mouth (n = 33, 69%) and fatigue (n = 23, 48%). Treatment mostly interfered with general activities. PROs were not affected by receipt of surgery; however, receipt of systemic therapy was associated with worse general activity (*P* = 0.044). Longitudinal analyses revealed mildly worse dry mouth (from 0 of 10 points to 1.5 of 10, *P* = 0.012) and numbness/tingling (from 0 of 10 to 0.5 of 10, *P* = 0.020).

**Conclusions:**

Ipsilateral neck RT for N2b tonsil SCC was associated with only a mild PRO symptom burden after at least 18 months, and therefore, a well-tolerated toxicity reduction strategy for appropriate patients.

## Introduction

Effective treatment of locally advanced head and neck (HN) cancer depends on the subsite but typically requires the use of multimodal regimens, including radiation therapy (RT), surgery, and systemic therapy such as chemotherapy. These modalities are associated with a wide array of treatment-related toxicities depending on the modality used. Toxicity is highly site-specific with respect to RT, and toxic effects can include fatigue, mucositis, xerostomia, dysgeusia, dysphagia, odynophagia, ototoxicity, osteoradionecrosis, cranioneuropathy, soft tissue fibrosis or necrosis, and carotid artery toxicity such as atherosclerosis, arterial stiffness, or arterioradionecrosis [1–7].

Treatment-related toxicities associated with various treatments have historically been determined by treating physicians. However, recognition is increasing regarding the importance of evaluating toxicities from the perspective of the patients in the comprehensive management of treatment-induced side effects in HN cancer patients. Patient-reported outcomes (PROs) have become increasingly valued in making these assessments, as they enable a more comprehensive evaluation of treatment outcomes and facilitate shared decision-making between patients and physicians. PROs, such as those obtained with the use of instruments such as the MD Anderson Symptom Inventory for Head and Neck Cancer (MDASI-HN), allow effective management strategies to be tailored to each patient. The MDASI-HN was developed to assess the incidence, severity, and burden of HN cancer patient symptoms as well as their interference with the patients' daily lives [8], and has been validated in several studies to identify patients with declining quality of life [9], to explore symptom burden in select patients with early-stage tonsil cancer [10], including older patients where most patients demonstrated mild-to-moderate symptoms [11], and to demonstrate shifts in the types of symptoms that were most severe during the course of RT in a longitudinal study of mostly oropharyngeal tumors [12].

Collectively, physician- and patient-reported toxicities from RT for HN cancer have prompted concerted efforts to de-intensify treatments while maintaining acceptable oncologic outcomes. Efforts to reduce RT-related toxicity are multifaceted and include improved RT delivery techniques (such as the use of intensity-modulated radiation treatment [IMRT], volumetric modulated arc therapy [VMAT], or proton beam therapy [13]), image-guided RT, dose reduction/altered fractionation [14], effective toxicity management, reduced dose to critical organs [15], de-intensification of systemic therapy [16], use of transoral robotic surgery followed by reduced-dose adjuvant RT [17], and RT to the unilateral/ipsilateral neck instead of the bilateral neck in some patients [18]. However, the use of unilateral neck RT is controversial because of the increased risk of contralateral neck failure, as demonstrated in a recently published systematic review and meta-analysis [19]. This approach is typically reserved for early-stage, well-lateralized faucial tonsillar human papillomavirus (HPV)-associated squamous cell carcinoma (SCC) with limited nodal involvement (typically 0–1 nodes, i.e., N0-N2a disease). The use of ipsilateral RT becomes even more controversial for patients with several involved ipsilateral nodes (i.e., N2b disease) because of an even higher risk of contralateral nodal failure.

Building on the excellent outcomes observed for patients with T1-2 N2b (per the 7th [2010] edition of the American Joint Committee on Cancer [AJCC]-7 staging manual) tonsillar cancer, a subset of our group’s publication on outcomes after unilateral RT [20], we conducted a multi-institutional retrospective study on the outcomes of N2b patients treated with unilateral RT, and we observed excellent locoregional control and overall survival with low risk of contralateral nodal failure [21]. Here, we report PROs from an updated cohort from our institution with N2b tonsil cancer treated with ipsilateral RT, including a subset with longitudinal follow-up, based on previously collected MDASI-HN scores.

## Materials and methods

### Patient population

After approval by the institutional review board for this retrospective observational study, we reviewed our internal database of patients treated from 2005 through 2018 with unilateral RT at our institution who completed the MDASI-HN survey at a minimum of 18 months after completion of treatment. At the initial consultation and follow-up visits, all patients underwent detailed head and neck examinations and baseline imaging with computed tomography (CT) or fluorodeoxyglucose (FDG) positron emission tomography (PET)/CT, and disease staging per AJCC-7. Patients with any evidence of disease at the time of enrollment or at survey completion were excluded. We selected patients with ≤ 4 cm, well-lateralized primary tonsillar SCC and multiple ipsilateral lymph nodes ≤ 6 cm, with or without extranodal extension (i.e., AJCC-7 T1-2 N2b). The Eastern Cooperative Oncology Group (ECOG) performance status scores, smoking status, and HPV/p16 status were determined from chart review. Eligible patients had provided informed consent and were offered the MDASI-HN questionnaire to complete, most of which were completed after a median of 28.9 months after RT (range 19–102 months).

### Treatment

Overall, treatment recommendations were determined at multidisciplinary conferences of radiation, surgical, and medical oncologists. Dose, fractionation, and target volumes were reviewed in radiation oncology quality assurance meetings. All patients received IMRT, except for two who received intensity-modulated proton therapy [22]. The target included the tonsillar region and neck above the arytenoids, matched to an anterior photon supraclavicular field that treats the lower ipsilateral neck by using a mono-isocentric technique. A dose range of 63–72 Gy was delivered to the planning target volume (PTV), which was generated as a 3-mm expansion of the clinical target volume, which, in turn, was created from an 8–10 mm volumetric expansion of the gross tumor volume of the primary tumor and/or the involved nodes [23]. PTV volumes of subclinical disease in the ipsilateral neck were covered with lower doses of approximately 54–60 Gy, depending on the dose to the tumor PTV and whether the patients had had simple tonsillectomy or trans-oral robotic surgery before RT or nodal dissection before RT. At the discretion of the medical oncologist, some patients received induction chemotherapy with a taxane, platinum, 5-fluorouracil, or a combination of the three, and/or concurrent cisplatin, carboplatin or cetuximab [24].

### MD Anderson symptom inventory for head and neck cancer

The MD Anderson Symptom Inventory for head and neck cancer (MDASI-HN) is a validated symptom burden questionnaire [8–12, 25, 26] with three component domains: (i) a core domain of 13 symptoms common in all cancer patients (i.e., pain, fatigue, nausea, disturbed sleep, distress/feeling upset, shortness of breath, difficulty remembering, lack of appetite, drowsiness, sadness, vomiting, numbness/tingling and dry mouth) (we considered ‘dry mouth’ to be a HN-related symptom); (ii) 9 HN cancer-specific domains (i.e., problems with mucus; swallowing/chewing; choking/coughing; voice/speech; skin pain/burning/rash; constipation; tasting food; mouth/throat sores; and teeth or gum soreness); and (iii) 6 interference domains (i.e., interference with general activity, mood, work [including housework], relations with others, walking, and enjoyment of life). The scores for items in the first two domains range from 0 (not present) to 10 (as bad as you can imagine), and the interference scores range from 0 (does not interfere) to 10 (interfered completely).

### Statistical analysis

Summary statistics were used to describe clinical, disease, and treatment characteristics. The symptom scores were analyzed by univariate analysis with either Wilcoxon or Mann–Whitney U test. Wilcoxon test was used for longitudinal analyses. Symptom scores between patients who underwent surgery before RT and those who did not were compared with Mann–Whitney U tests. Symptom scores among patients who did or did not receive systemic therapy were compared with the Mann–Whitney U test. *P* ≤ 0.05 was considered statistically significant. All statistical analyses were performed with GraphPad Prism version 9.2.0.

## Results

### Patient characteristics

Forty-eight eligible patients had completed the MDASI-HN at ≥ 18 months after RT. Patient and treatment characteristics are summarized in Table 1. The median age at diagnosis was 55 years (range, 29–71 years), and 13 patients (27%) were female. All patients had good performance status (ECOG scores of 0–1). High-risk HPV or its p16 surrogate was detected in 36 patients (75%), and 11 (23%) had unknown HPV status. Thirty-three patients (69%) had T1 disease, and 15 (31%) had T2 disease. All 48 patients had N2b disease with level II node involvement, 18 (37%) had level III, and 4 (8%) had level IV involvement; no patient had level V involvement. Twenty patients (42%) had at least two involved nodal levels, and 15 (31%) had extranodal extension detected only clinically (3 patients), only radiographically (3 patients), both clinically and radiographically (1 patient), pathologically (7 patients) and both clinically and pathologically (1 patient). Most patients were treated with IMRT. The radiation treatment targets included ipsilateral tonsil cancer and ipsilateral hemi-neck. The median RT dose was 66 Gy (range 63–72 Gy). Nine patients (19%) received induction chemotherapy consisting of cisplatin, docetaxel, paclitaxel/carboplatin, paclitaxel/carboplatin/cetuximab, or docetaxel/cisplatin/fluorouracil, and 22 (46%) received concurrent chemotherapy (cisplatin, carboplatin, or cetuximab). Twenty-seven patients underwent surgery before RT: 21 (44%) underwent simple tonsillectomy, and 6 (12%) underwent transoral robotic surgery.

**Table 1:** Baseline patient and treatment characteristics

Characteristic	Value or no. (%)
Median age, years (range)	55 (29–71)
Sex	
Female	13 (27)
Male	35 (73)
ECOG performance status score	
0	35 (73)
1	13 (27)
Smoking status	
Current	3 (6)
Former	17 (35)
Never	28 (58)
HPV/p16 status	
Positive	36 (75)
Negative	1 (2)
Unknown	11 (23)
T Category, AJCC-7	
1	33 (69)
2	15 (31)
No. of nodal levels involved	
1	28 (53)
2	18 (37)
3	2 (4)
Extranodal extension	
Yes	15 (31)
No	32 (67)
Unknown	1 (2)
Systemic therapy	
Induction	9 (19)
Concurrent	22 (46)
Both	1 (2)
None	16 (33)
Pre-radiation surgery	
Simple tonsillectomy	21 (44)
TransOral robotic surgery	6 (12)
None	21 (44)

### MDASI-HN domains

**Fig. 1:**
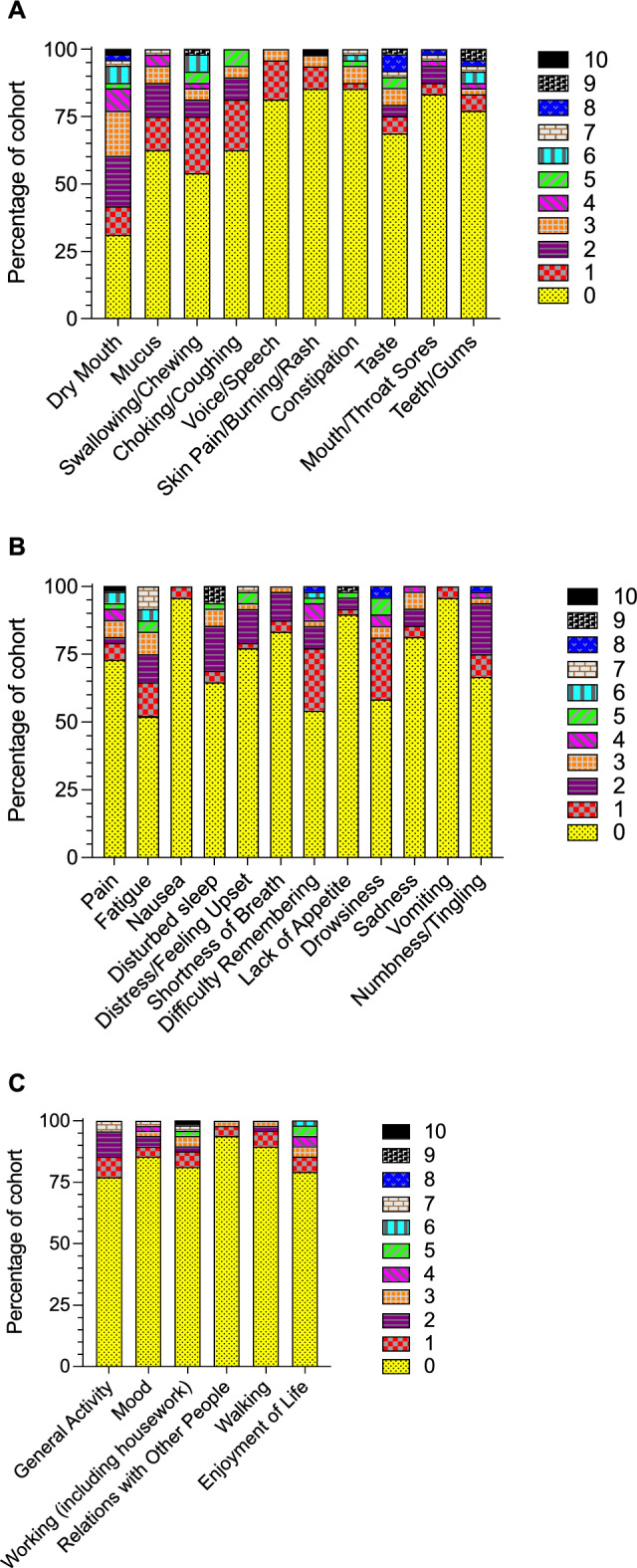
Distributions of MDASI-HN patient-reported outcomes. Patient-reported scores for the **A** MDASI-HN domain **B** Core domain, and **C** Interference domain. Colors indicate severity scores from 0 (not present) to 10 (as bad as you can imagine). MDASI-HN, MD Anderson Symptom Inventory—Head and Neck Cancer

Dry mouth, reported by 33 patients (69%), was the most commonly reported HN-related symptom; in contrast, the symptoms reported least often (by seven patients each, 15%) were skin pain/burning/rash and constipation from narcotics for pain control (Fig. 1A). More than 50% of those who reported other symptoms reported no MDASI-HN-related symptoms. One patient reported a 10/10 symptom severity for both dry mouth and skin pain/burning/rash.

### MDASI core domains

At least 25 participants (52%) reported no symptoms in any of the 13 MDASI core domains (Fig. 1B). Among the reported symptoms, “fatigue” was most commonly reported (23 patients, 48%) and “nausea” and “vomiting” were the least frequently reported (2 patients, 4%). Only one patient reported 10/10 pain symptoms, and this was the same patient with 10/10 symptom scores for both dry mouth and skin pain/burning/rash.

### MDASI interference domains

Over thirty-six (≥ 77%) participants reported no interference symptoms (Fig. 1C). Among those who reported symptoms that interfered with their lives, “general activity” was most common (11 patients, 23%) and “relations with other people” was the least frequently reported (3 patients, 6%). A different patient than the one mentioned above reported a 10/10 score for interference with work, including housework.

### Association with additional treatment

**Fig. 2:**
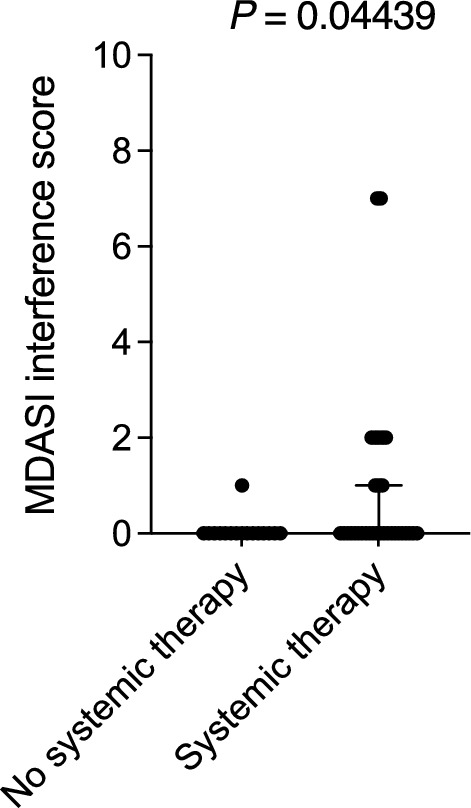
Association of general activity scores of MDASI—interference domains with the administration of systemic therapy**.** Data from 16 patients who did not receive systemic therapy and 32 patients who received induction chemotherapy, concurrent chemotherapy, or both are shown. Error bars represent medians with interquartile ranges. MDASI, MD Anderson Symptom Inventory. *P* ≤ 0.05 indicates a statistically significant difference

Receipt of surgery was not significantly associated with the distribution of PRO scores for any of the three MDASI domains (data not shown). However, the use of systemic therapy was associated with worse scores for only “General Activity” (*P* = 0.04439; Fig. 2).

### Longitudinal changes

**Fig. 3:**
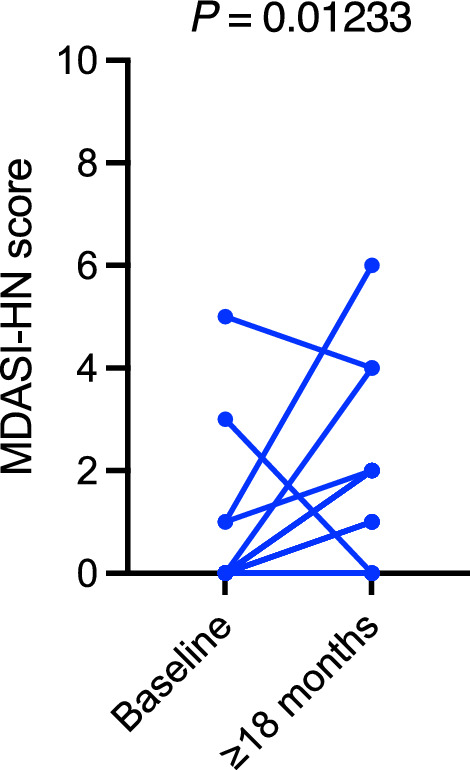
Longitudinal changes in dry mouth scores of the MDASI-HN domains. Data pairs of 18 patients from baseline to at least 18 months after radiation therapy are shown and connected with stems. MDASI-HN, MD Anderson Symptom Inventory—Head and Neck Cancer. *P* ≤ 0.05 indicates a statistically significant difference

**Fig. 4:**
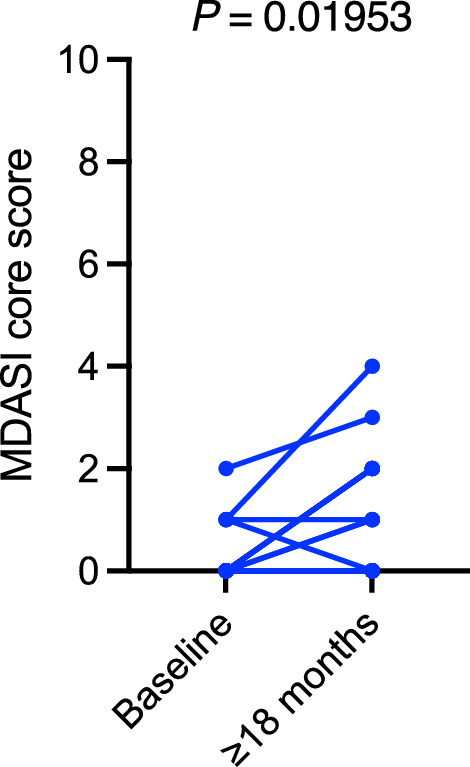
Longitudinal changes in numbness and tingling scores of the MDASI core domains. Data pairs of 18 patients from baseline to at least 18 months after radiation therapy are shown and connected with stems. MDASI, MD Anderson Symptom Inventory. *P* ≤ 0.05 indicates a statistically significant difference

Baseline scores for the MDASI-HN were available for 18 patients, and longitudinal comparisons showed no statistically significant changes from the pretreatment baseline to at least 18 months after RT across most symptom domains. However, the median scores worsened slightly for dry mouth (from 0/10 to 1.5/10, *P* = 0.01233; Fig. 3) and numbness/tingling (from 0/10 to 0.5/10, *P* = 0.01953; Fig. 4).

## Discussion

Patients with early-stage faucial tonsillar SCC have excellent survival; therefore, it is crucial to reduce the incidence of acute and late treatment-related toxicities. We previously showed that limiting the RT treatment volume to the primary site and unilateral hemi-neck led to excellent 5-year rates of overall survival and locoregional control, with very few contralateral neck recurrences [20, 21]. Here, we report that the incidence of treatment-associated toxicity from the patients’ perspective using the MDASI-HN is minimal, regardless of whether they underwent surgery for diagnosis or treatment.

PROs are increasingly being integrated into oncology decision-making, including in HN cancer [27, 28]. A meta-analysis of 138 studies from 2013 through 2018 [29] indicated that PROs provide independent prognostic information and seem to correlate with clinician-reported outcomes. McDowell et al. reported similarities between toxicities documented by physicians and those reported by patients with HPV^+^ SCC of the tonsils treated with either unilateral or bilateral IMRT [30]. Moreover, the MDASI-HN, including the core and interference domains, has been validated in numerous studies [8–12, 26, 31], and has been shown to be predictive of objective Common Terminology Criteria for Adverse Events in patients with HN cancer [25]. In addition to the MDASI-HN, the MD Anderson Dysphagia Index (MDADI) is also used to assess patient-reported dysphagia burden. Our collaborators recently demonstrated no apparent differences in dysphagia among patients with tonsillar SCC who underwent unilateral RT, bilateral RT, or transoral robotic surgery based on both physician-graded and patient-reported scores [32].

The most common MDASI-HN symptom in our study was very mild dry mouth (n = 33, 69%). The minimal increase from baseline (0/10) to ≥ 18 months after RT (1.5/10) showed that late xerostomia was negligible after unilateral RT. However, patients who received systemic therapy, whether induction or concurrent, were also at risk for peripheral neuropathy (numbness/tingling) and interference with general activity. These findings are consistent with previous findings of favorable PROs in patients with early-stage tonsillar cancer treated with IMRT to either the ipsilateral or bilateral neck [10] and the largely favorable long-term PROs in ≥ 65-year-old patients with oropharyngeal cancer (41% tonsillar primaries) treated with definitive RT [11]. The most commonly reported symptom of dry mouth in the current study was also consistent with a previous report [10]. We suspect that this symptom most likely arose from the RT dose to the submandibular gland, as the dose constraints for this structure were less stringent until 2011, when Murdoch-Kinch et al. showed a dose-dependent exponential reduction in submandibular gland function and improvement over time if a submandibular gland mean dose constraint of ≤ 45 Gy was met [33]. Complete avoidance of the ipsilateral major salivary glands is challenging because of the proximity of the parotid and submandibular glands to the tonsils and level II nodes, which were irradiated in all patients.

Our findings have significant clinical implications especially in patients with HPV-related HN SCC as they have good prognosis. HN RT is one of the most challenging RT for patients to undergo due to multiple, concurrent and potentially severe side effects. The most consequential acute side effect in most cases is dysphagia that is compounded by RT-induced increased secretions, pain, xerostomia and dysgeusia. As a result, patients often get dehydrated, requiring repeated intravenous fluid administrations, and sometimes lose significant weight, requiring feeding tube insertion. These additional interventions further affect the abilities of patients to complete treatment as well as their qualities of life. As a result, being able to reduce the treatment volume without compromising tumor control outcomes, and consequently, minimizing the risk of developing these side effects is critical. While ipsilateral neck RT is a commonly accepted approach in patients with HPV-related well-lateralized tonsillar SCC, that approach is not as widely accepted for N2b patients. Although some of the patients in this report have non-HPV-mediated tumors, the general observation of mild PROs with mostly xerostomia suggests that the use of volume reduction should be considered as a reasonable method of reducing toxicity in N2b patients.

Our study had several limitations. First, this was a single-arm retrospective study, which was prone to bias because the treatments were not blinded. A randomized controlled trial with a comparison group of patients who received bilateral neck RT would yield more compelling findings. Second, some patients received surgery or chemotherapy in addition to RT, and chemotherapy was induction-only in some patients and concurrent in others. Third, relatively few patients met the inclusion criteria of having N2b disease at diagnosis but being disease-free and completing the MDASI-HN questionnaire at least 18 months after RT. Notably, the limited number of patients available for baseline and longitudinal MDASI-HN data in this study makes it difficult to estimate the acute symptom burden and its immediate trajectory after unilateral neck RT. However, since symptom burden scores are expected to increase after RT, the minimal to zero MDASI-HN scores in patients who had completed treatment at least 18 months previously suggests that the long-term symptom burden is low after unilateral neck RT. Longitudinal analyses among the 18 patients with baseline scores did not reveal significant increases in symptom burden scores compared with baseline, except for worse dry mouth consistent with radiotherapy stigmata and worse numbness/tingling that could be attributed to concurrent chemotherapy. A larger sample size would presumably provide more generalizable data, although our data were consistent and without large variations among patients with AJCC 7 N2b disease. Finally, a much longer follow-up study is warranted to identify the development of late toxicities.

In summary, our findings show that RT limited to the ipsilateral neck in patients with well-lateralized HN primary tumors and multiple involved lymph nodes (≤ 6 cm), regardless of extranodal extension status, not only yielded excellent oncologic outcomes with minimal contralateral failures [20, 21] but also had minimal effects on long-term functionality and comfort from the patients’ perspective, as measured by the MDASI-HN. The observation that dry mouth was the most commonly reported symptom across all domains supports the need to investigate management strategies to permanently preserve salivary gland function. With a few exceptions, the severity of the patient-reported toxicities was minimal, indicating that most of these potentially cured patients continue to have good quality of life without chronic adverse effects.

## Data Availability

Data is provided within the manuscript or available upon reasonable request from the corresponding author.

## Abbreviations

ECOG: Eastern Cooperative Oncology Group
HPV: Human papillomavirus
AJCC-7: 7Th (2010) edition of the American Joint Committee on Cancer staging manual
